# P-1535. Evaluation of *Enterobacterales* Bacteremia with Piperacillin/tazobactam Minimum Inhibitory Concentrations of 16 mcg/mL: Demystifying the Susceptible-dose Dependent Breakpoint

**DOI:** 10.1093/ofid/ofae631.1703

**Published:** 2025-01-29

**Authors:** Ryan Shou, Thien-Ly Doan, Barbara Kamel, Santosh Dahal, Aya Haghamad, Stefan Juretschko, Henry Donaghy

**Affiliations:** Northwell - Long Island Jewish Medical Center, New Hyde Park, New York; Long Island Jewish Medical Center, New Hyde Park, New York; Northwell Health, New Hyde Park, New York; Northwell - Long Island Jewish Medical Center, New Hyde Park, New York; Northwell Laboratories, Lake Success, New York; Northwell Health laboratories, Little Neck, New York; Northwell Health, New Hyde Park, New York

## Abstract

**Background:**

CLSI updated piperacillin/tazobactam (pip/tazo) MIC breakpoints for *Enterobacterales* to 16 mcg/mL for susceptible dose dependent (SDD) and recommends 4.5 g IV q8h over 4 hours. This study aims to determine the usage and clinical outcomes of pip/tazo versus other antibiotics for *Enterobacterales* bacteremia with SDD. The primary outcome is treatment failure, defined as a composite of in-hospital mortality; persistent fever or leukocytosis on day 3; microbiological failure on day 3-5; and microbiological relapse on day 5-30. Secondary outcomes include hospital length of stay (LOS), hospital readmission, and time to clinical resolution of infection.

**Figure:**
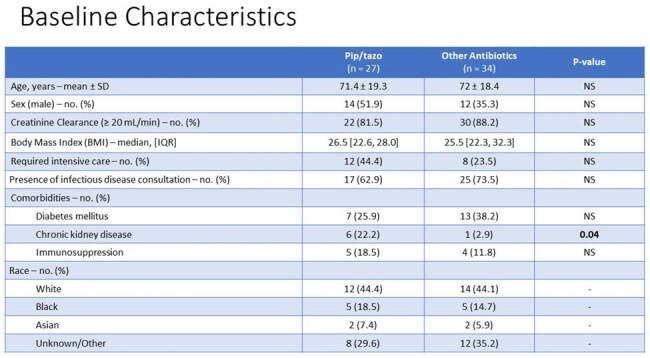

The baseline characteristics were well-matched and the patient population consisted mostly of elderly patients with normal renal function who require some intensive care. And a majority of both groups had ID consultation. As for other characteristics, diabetes was the most common comorbidity encountered and there was a difference detected in terms of chronic kidney disease with pip/tazo having more patients than other antibiotics and 44% of patients in both groups were white.

**Methods:**

This IRB-approved, retrospective descriptive study evaluated admitted adults at a Northwell Health hospital from 1/2019 to 10/2023, with positive blood cultures for *Enterobacterales* with a pip/tazo MIC of 16. Patients were excluded if the isolate was an ESBL strain, resistance to pip/tazo, or if they expired or were discharged within 48 hours of antibiotic initiation. Data collection included demographics, microbiology isolates, antibiotic regimens, and outcomes.

Reasons for Pip/tazo Conversions

**Figure:**
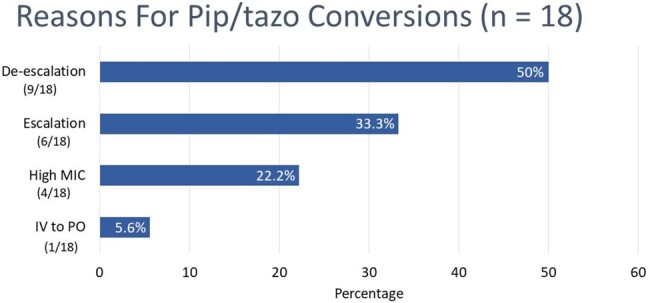

As for the reasons for conversions from pip/tazo to another agent(s), a majority were de-escalation or escalation. Only 4 patients or 22% had documentation of high MIC as the reason for change.

**Results:**

A total of 80 patients were screened, of which 61 were included in the study. The pip/tazo group had 27 patients, while the other antibiotic group included 34. Most common source of bacteremia was urinary (63.9%), followed by intra-abdominal (19.7%). *E. coli* was most common (82%) followed by *Serratia marcescens* (18%). The majority of patients received 3.375 g (88.9%) and only 11.1% received 4.5 g. Composite treatment failure was no different between the 2 groups. Although not statistically significant, pip/tazo group had a longer LOS (8 vs. 6 days) increased time to clinical resolution of infection (median 3 vs. 2 days) and similar re-admissions (18.5% vs 23.5%).

Results - Primary and Secondary Outcomes

**Figure:**
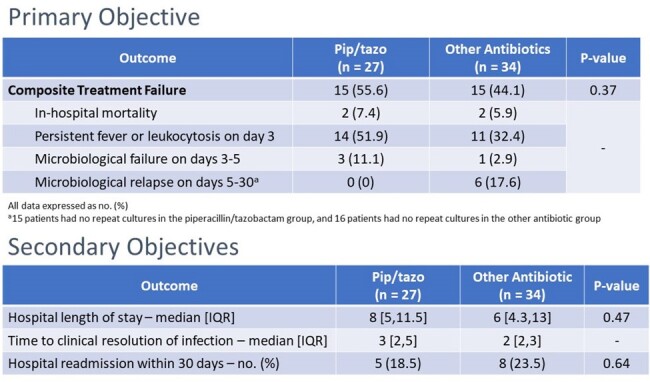

The composite outcome of treatment failure, both group had 15 patients who failed with a p value of 0.37. Hospital mortality and microbiological failure on day 3-5 were similar with each group, pip/tazo had more patients with persistent leukocytosis while other antibiotic group had 6 patients in the microbiological relapse. As for the secondary objectives, both groups had similar LOS and hospital readmission which were not statistically significant,and numerically, there seem to be no difference for time to clinical resolution of infection, however the hospital length of stay for pip/tazo was greater by 2 days.

**Conclusion:**

No differences were found in treatment failure, time to clinical resolution and hospital readmission between groups. Longer LOS was seen with pip/tazo which can have a financial impact. Higher dosing of pip/tazo did not impact clinical outcomes. Study limitations included a small sample size, retrospective chart review, and varying practices among the different sites. More studies are needed to evaluate the usage of pip/tazo in *Enterobacterales* with high MIC of 16.

Subgroup Analysis of Piperacillin/tazobactam

**Figure:**
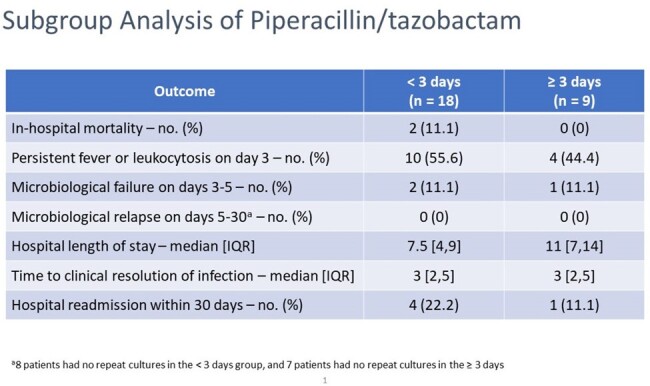

This is a subgroup analysis of patients on pip/tazo split into less than 3 days or 3 days or more of therapy. Most outcomes are similar except that the greater than or equal to 3 days had a longer median length of stay compared to less than 3 days. The less than 3 days group had more patients with persistent fever or leukocytosis than the 3 days or greater treatment arm.

**Disclosures:**

**All Authors**: No reported disclosures

